# Correlating MRI‐based brain volumetry and cognitive assessment in people with Down syndrome

**DOI:** 10.1002/brb3.3186

**Published:** 2023-07-26

**Authors:** Osama Hamadelseed, Thomas Skutella

**Affiliations:** ^1^ Department of Neuroanatomy, Institute of Anatomy and Cell Biology University of Heidelberg Heidelberg Germany

**Keywords:** cognition, Down syndrome, MRI, neuroanatomy

## Abstract

**Introduction:**

Down syndrome (DS) is the most common genetic cause of intellectual disability. Children and adults with DS show deficits in language performance and explicit memory. Here, we used magnetic resonance imaging (MRI) on children and adults with DS to characterize changes in the volume of specific brain structures involved in memory and language and their relationship to features of cognitive‐behavioral phenotypes.

**Methods:**

Thirteen children and adults with the DS phenotype and 12 age‐ and gender‐matched healthy controls (age range 4–25) underwent an assessment by MRI and a psychological evaluation for language and cognitive abilities.

**Results:**

The cognitive profile of people with DS showed deficits in different cognition and language domains correlating with reduced volumes of specific regional and subregional brain structures, confirming previous related studies. Interestingly, in our study, people with DS also showed more significant parahippocampal gyrus volumes, in agreement with the results found in earlier reports.

**Conclusions:**

The memory functions and language skills affected in studied individuals with DS correlate significantly with the reduced volume of specific brain regions, allowing us to understand DS's cognitive‐behavioral phenotype. Our results provide an essential basis for early intervention and the design of rehabilitation management protocols.

## Graphical abstract

The study investigated the neuroanatomy and neuropsychology of Down syndrome using neuroimaging and psychological assessments. The cognitive profile of individuals with Down syndrome revealed deficits across various cognitive and linguistic domains correlated with smaller volumes of regional and subregional brain structures. The study's findings help us comprehend the cognitive‐behavioral phenotype of people with Down syndrome and offer a crucial foundation for developing rehabilitation management protocols and early intervention strategies.

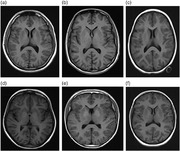

## 1 INTRODUCTION

Down syndrome (DS), the most frequent genetic cause of intellectual disability, affects approximately one in every 700 live births (Parker et al., 2010). Intellectual disability is the most well‐known feature of the DS cognitive‐behavioral phenotype (Gibson, 1978). However, research has indicated a profile of relative strengths and weaknesses that characterizes children with DS compared to younger, typically developing peers matched on the developmental level and same‐age peers with other forms of intellectual disability (Fidler et al., 2009). Those with DS have language functioning deficits in several domains that exceed their overall cognitive limitations. Furthermore, difficulties with explicit memory are more prevalent than global impairments (Hamner et al., 2018; Jarrold et al., 2007).

Many in vivo magnetic resonance imaging (MRI) investigations have defined the brain change pattern underlying DS (Aylward et al., 1999; Aylward, Habbak, et al., 1997; Aylward, Li, et al., 1997; Jernigan et al., 1993; Kates et al., 2002; Kesslak et al., 1994; Pearlson et al., 1998, 1990; Pinter, Brown, et al., 2001; Pinter, Eliez, et al., 2001; Raz et al., 1995; Schapiro et al., 1989; Śmigielska‐Kuzia et al., 2011; Weis et al., 1991; White et al., 2003). The primary findings from these studies showed that DS has a link to decreased total brain volume (Jernigan & Bellugi, 1990; Kates et al., 2002; Pearlson et al., 1998; Pinter, Eliez, et al., 2001; Śmigielska‐Kuzia et al., 2011), as well as specific reductions in cerebellar (Jernigan & Bellugi, 1990; Pinter, Eliez, et al., 2001, White et al., 2003; Raz et al., 1995) and hippocampal volumes (Pearlson et al., 1998; Pinter, Eliez, et al., 2001, White et al., 2003; Raz et al., 1995; Śmigielska‐Kuzia et al., 2011). Decreases in the frontal and temporal lobes have also been reported (Frangou et al., 1997; Kates et al., 2002; Raz et al., 1995; Śmigielska‐Kuzia et al., 2011; White et al., 2003).

In contrast, other studies observed increased parahippocampal gyrus volumes (Kesslak et al., 1994; Raz et al., 1995; White et al., 2003) and preserved parietal lobar gray matter volumes (Pinter, Eliez, et al., 2001). However, these studies have limits due to low resolution, small group size, and limited age range. Additionally, they used traditional region‐of‐interest volumetry. It is susceptible and can only identify volumetric changes in specific brain areas manually or semiautomatically demarcated on MR images. Recent investigations applied voxel‐based morphometry. This wholly automated approach involves voxel‐by‐voxel analysis of the entire brain in spatially normalized MR images (Carducci et al., 2013; Menghini et al., 2011; Rigoldi et al., 2009; Teipel et al., 2004; White et al., 2003). This method can detect structural anomalies on a global scale (Ashburner & Friston, 2000; Wright et al., 1995). Among these investigations, only one study by Menghini et al. (2011) explored the relationship between the brain and cognitive behavior in adults with DS. The limitation of this study was that it did not only involve adults with DS. Nevertheless, it did not include all related brain regions and essential language and memory domains, and its results were considered preliminary (Menghini et al., 2011).

A recent retrospective analysis of MRI examinations of DS participants (aged 0–22) and a cohort of neurotypical participants (aged 0–32) by McCann et al. (2021) reported different brain abnormalities. However, this study represents a sizeable neuroimaging study of DS. Nevertheless, it has limitations, such as the retrospective nature of the study, the lack of neurocognitive function assessments of people with DS (e.g., intelligence quotient [IQ] data), the lack of detailed patient interviews with complete comorbidity assessments, and the imbalanced pool of participants (McCann et al., 2021). An in vivo fetal and neonatal MRI assessment by Patkee et al. (2020) detected early alterations in cortical and cerebellar regional brain growth in DS in the second and third trimesters. Although this study allows us to detect early biomarkers that may predict the degree of cognitive deficits of DS in the early stages of life, it does not provide enough evidence for abnormal brain development without cognitive assessments (Patkee et al., 2020).

Lee et al. (2015) described dissociations in the thickness and surface area of the cortex in young people with DS. Specifically, they observed significant surface area reductions alongside increases in cortical thickness at the global and vertex levels relative to the sex‐ and age‐matched control group. Additionally, this study has limitations regarding not including neuropsychological examinations to study the brain–behavior relationship (Lee et al., 2015).

Using a developmental approach, a better understanding of the developing brain with DS will illuminate important neurological principles of DS in children and shed light on the foundations of adult phenotypes, particularly the increased risk of early Alzheimer's disease (AD) (Lee et al., 2015; Zigman & Lott, 2007). Furthermore, investigating DS helps us study the early stage of AD, as DS can provide a suitable pattern for understanding AD pathology and clinical features at an early age (Delabar et al., 1990; Hamadelseed et al., 2022; Hartley et al., 2015; Lott & Head, 2019; Mann, 1988).

Thus, the current study seeks to fill this gap and provide new data on structural neuroimaging of children with DS compared to children with normal brain development and cognitive‐behavioral phenotypes. These deficits in DS‐related research are due to problems attributable to the successful execution of development‐oriented neuroimaging. However, it is still unclear why previous reports did not adopt a similar method for neuroimaging children with DS as in other related neurodevelopmental disorders (e.g., fragile × syndrome, Williams's syndrome, and microdeletion syndrome 22q11), although such studies used modern imaging technologies and analyses and could expect common difficulties with imaging such groups (Hamner et al., 2018). To fill this gap, the need for a boost in increased neuroimaging research, mainly focusing on early childhood, using advanced neuroimaging techniques and best practice guidelines for neuroimaging in children, is expanding (Raschle et al., 2012).

Our study depends on the hypothesis that children and adults with DS have language and memory problems as they age. These problems are related to brain development, particularly neuroanatomical changes associated with these behavioral changes (Edgin, 2013). To evaluate these hypotheses, we will examine the relationship between different brain regions and cognitive scores for memory and language in children and adults with DS.

We will examine brain areas such as the temporal lobes, parietal lobes, and hippocampus that are entirely and subregionally involved in memory and language skills. Our study will analyze the volumes of the hippocampal areas involved in explicit memory deficits (dentate gyrus, Ammon's horn, and subiculum). Furthermore, we will consider structures such as the superior temporal gyrus (STG) and temporoparietal junction structures underlying impaired speech performance (e.g., angular gyrus, supramarginal gyrus, and occipitotemporal structures [e.g., fusiform gyrus]) (Friederici & Gierhan, 2013; Hickok & Poeppel, 2004) and characterize them in more detail using neuroimaging with volume analysis. We will use high‐resolution MRI acquisition techniques and advanced segmentation and image processing protocols to obtain more accurate quantitative image data in children with DS. We will study brain areas that previous studies have not considered, such as some regions in the temporal and parietal lobes or not adequately investigated as hippocampal and parahippocampal gyrus areas. By studying these areas of interest and comparing their volumes with the psychological results, we can approach language and memory problems in people with DS and provide a basis for further research, including early interventions.

There is a growing emphasis on diversity and inclusion in clinical and translational research study cohorts. Researchers recognize the importance of including individuals from diverse socioeconomic backgrounds, races, and ethnicities to obtain a more comprehensive understanding of various health conditions and ensure that research findings apply to a broader population.

This emphasis on diversity and inclusion is especially relevant for individuals with DS and intellectual disabilities. Historically, research studies involving individuals with DS have predominantly focused on cohorts from Europe and America. However, there is a need to expand the scope of research and include individuals with DS from diverse cultural and linguistic backgrounds. A recent consensus statement by Fidler et al. (2022) underscores the importance of including individuals with DS and intellectual disabilities from various backgrounds, including those from African nations with Arabic as the primary language. The present study could contribute significantly to this evolving field by showcasing a largely age‐matched, sex‐matched cohort of children with and without DS from an African nation where Arabic is the primary language. This approach provides a unique and valuable context to the findings, highlighting the similarities observed in MRI and cognitive studies of children with DS from Europe and America (Pinter, Eliez, et al., 2001, Windsperger & Hoehl, 2021). By emphasizing the diversity and inclusion of individuals with DS from different cultural and linguistic backgrounds, the study can be one of the first or few studies to shed light on this critical topic.

## 2 MATERIALS AND METHODS

### 2.1 Participants

We recruited our DS participants from DS care centers in Sudan and selected a control group from our DS participants’ family members and hospital records. Thirteen children and adults with DS (eight males and five females, mean age = 15 years, SD = 5.9, and range = 6.0–25) and 12 healthy controls (8 males and 4 females, mean age = 14 years, SD = 6.8, and range = 4.0–25) underwent MRI scans at Aliaa Specialist Hospital. DS participants and control persons had an assessment for IQ, including working memory, by applying the IQ test (the Stanford–Binet scale, the fifth picture of intelligence) and for language by using a language test (Luttas language development test scale) by a consultant medical psychologist and speech rehabilitation specialist at the Women & Child Health Development Organization. A pediatrician assessed people with DS clinically to exclude cardiac defects and any contraindications for sedation. The diagnosis of DS was established at birth or in early infancy and childhood by clinical examination with one karyotype diagnosis case.

### 2.2 Inclusion and exclusion criteria for the participants

We included only participants with a confirmed diagnosis of DS through medical records or genetic testing. We selected only typically developing people without any known neurological condition for control. In both groups, we excluded participants with significant comorbidities or neurological disorders (other than DS). We did not define any specific age ranges, cognitive functioning levels, or medication usage for inclusion or exclusion in our study. We tried to ensure that these criteria are ethically sound and do not exclude individuals without justifiable reasons. We aimed to make a balance between representative sampling and feasibility.

### 2.3 Sample size calculation

We conducted our study in cooperation with DS care centers in Sudan, including 250 children and adults with DS. Based on previous research and clinical knowledge, we decided to include 10% of them in the study, which means 25 participants. However, of these 25 participants, only 13 fulfilled all the inclusion criteria we established. So, we have 13 eligible participants to include in your study.

It is important to note that the decision to include only 10% of the people with DS enrolled in the care centers may have implications for the generalizability of our study findings. By selecting a smaller percentage of the population, we are narrowing our study's representation of individuals with DS. This could limit our findings’ generalizability to the broader population of individuals with DS.

It is crucial to consider the limitations imposed by the available resources, time constraints, and practical considerations when determining our study's final sample size.

### 2.4 Imaging

MRI is a radiographic sectional imaging technique that allows imaging different body levels with high soft‐tissue contrast. In contrast to computed tomography or conventional X‐ray imaging, MRI is based on a different magnetization of the body by the magnetics of MR tomography. MRI does not include X‐rays so that children can obtain a safe examination. MRI is a very patient‐friendly method that generally requires no special preparation. With the latest generation devices using a magnetic field strength of 1.5 Tesla (T) and 3.0 T, the entire body can receive the examination in less than 20 min. The most used MR scanners are 1.5 T (Tesla) or 3 T systems. Both systems allow the quantification of global and regional brain structures.

MR scans on 1.5 T systems are sufficient to quantify relatively small brain structures such as the hippocampus (Keller & Roberts 2009). In Sudan, a 3D structure MRI technique with a 1.5 T Siemens scanner is common in Khartoum hospitals. 3D Slicer 2.6 is available for volume measurement.

Participants had MRI scans using a 1.5 Tesla Siemens Syngo MR system at the Radiology and Medical Imaging Department, Aliaa Specialist Hospital. Some children have sedation to be stable and restrained from movement.

### 2.5 Measurement and segmentation of total brain volume and hippocampal volume

To measure the volumes of different brain regions, we used the volBrain online system (http://volbrain.upv.es). This online MRI brain volumetry system briefly provides free automated brain analysis and segmentation for various brain structures with accurate and detailed results. To use this system, users should first register by providing personal information such as the email address, name, and name of the institution to which they belong. The user submits a single anonymized compressed MRI T1w Nifti file in a web interface to analyze the imaging data, and the web server accepts requests. After approximately 12 min, the results will be ready, downloaded as a pdf file, and received via email (Manjón & Coupé, 2016). We used two pipelines available on the online system to measure our data: (1) the HIPS pipeline, which is a pipeline for automatic hippocampal subfield segmentation from monospectral (T1) images using the Kulaga‐Yoskovitz et al. (2015) segmentation protocol and (2) the vol2Brain pipeline, which provides automatic brain segmentation dividing the volume into 135 structures. It also provides tissue, macrostructure, lobe segmentation, and cortical thickness. Researchers compared this brain volumetry system with other software packages that segment the subcortical brain. They found it more reproducible and accurate. Therefore, it can be considered one of the first few platforms that offer hippocampal segmentation, which will help diagnose and study AD (Manjón & Coupé, 2016).

### 2.6 Neuropsychological assessment

We assessed DS participants and controls with the Stanford–Binet intelligence scales, fifth edition (SB5), to measure memory and cognitive abilities. Additionally, we applied the Luttas language development test scale to measure different language development scales among DS samples and healthy controls. Considering the community standards and environment, we selected these tests to comply with the study's goal of correlating brain area anatomy, cognitive status, and language abilities. Some children with special needs cannot acquire all the skills and abilities to respond to the applied test early. These selection criteria and limitations might explain the failure to include children under 5 years in this study, as they do not have enough skills to continue with the tests.

### 2.7 Stanford–Binet intelligence scale, fifth edition (SB5)

The Stanford–Binet occurred as an intelligence test in 1916. The recent version (published in 2003) is the Stanford–Binet intelligence scales, fifth edition (SB5), a self‐administered intelligence and cognitive abilities test for people aged 2–85. SB5 provides early childhood assessments and is valuable for psychoeducational purposes, plans, and later career development to detect various developmental disorders (Roid & Pomplun, 2012). It is an IQ test that measures five cognitive abilities in nonverbal and verbal formats: fluid reasoning, knowledge, quantitative reasoning, visuospatial processing, and working memory (see Tables 1 and 2). Because it is effective with many diverse groups independent of gender, race, culture, religion, area, or socioeconomic level, the SB‐5 is considered one of the most extensively utilized intelligence tests.

**TABLE 1:** Univariate summary statistics of the measured variables of the control group.

Variable	*N*	Mean	Median	SD	IQR	Minimum	Maximum
Age	12	14.00	13.000	6.88	9.7500	4.00	25.00
Total brain	12	1028.74	958.100	210.87	283.6275	792.70	1354.13
White matter	12	404.44	390.510	80.60	69.5575	259.19	584.34
Grey matter	12	624.30	593.890	155.82	227.8075	433.09	882.70
Cerebrum	12	914.98	858.420	192.25	240.9025	693.88	1216.18
Cerebellum	12	105.76	108.210	22.80	28.5950	71.06	148.36
Brainstem	12	15.42	14.230	3.54	5.2050	11.53	21.10
Hippocampus	12	5.53	5.735	1.17	1.5275	3.48	6.97
Dentate gyrus	12	.25	.220	.14	.1600	.07	.52
Ammon's horn	12	3.53	3.735	.86	1.1025	2.06	4.69
Subiculum	12	1.75	1.685	.36	.3275	1.29	2.65
Frontal lobe	12	169.94	175.005	48.81	71.4675	101.31	253.87
Parietal lobe	12	89.60	92.940	32.62	41.3225	49.20	157.52
Angular gyrus	12	19.49	20.560	4.76	6.0825	12.53	29.27
Supramarg. gyrus	12	13.66	11.785	5.25	7.3250	7.48	23.60
Temporal lobe	12	89.48	86.340	23.25	35.7300	61.61	136.09
Fusiform gyrus	12	15.59	14.620	5.30	7.1375	7.60	25.52
*S*. temp. gyrus	12	10.84	8.360	5.86	8.8950	4.19	22.98
Occipital lobe	12	69.74	66.685	18.55	22.1675	43.43	103.51
Parahippoca. gyrus	12	5.34	5.325	1.84	2.4175	2.73	8.91
Total IQ	12	101.75	104.000	13.07	18.7500	86.00	113.00
Nonverbal IQ	12	99.75	101.500	8.88	10.2500	88.00	108.00
Verbal IQ	12	103.75	105.000	16.52	23.7500	85.00	120.00
Working memory	12	96.00	95.000	18.38	22.5000	76.00	118.00
Total language	12	94.62	95.200	34.05	27.4750	52.60	135.50
Expressive language	12	123.25	140.900	52.22	34.2000	46.80	164.40
Receptive language	12	111.80	118.800	45.94	44.2000	50.60	159.00
Fluid reasoning	12	102.00	105.000	8.12	4.5000	90.00	108.00
Knowledge	12	114.00	114.000	19.60	12.0000	90.00	138.00
*Q*. reasoning	12	98.25	100.000	14.64	13.2500	79.00	114.00
*V*. *S*. processing	12	99.75	97.500	15.84	23.7500	86.00	118.00

Abbreviations: IQ, intelligence quotient; IQR, interquartile range; *N*, the number of studied samples; parahippoca. gyrus, parahippocampal gyrus; *Q*. reasoning, quantitative reasoning; *S*. temp. gyrus, superior temporal gyrus; SD, standard deviation; supramarg. gyrus, supramarginal gyrus; *V*. *S*. processing, visuospatial reasoning.

**TABLE 2:** Univariate summary statistics of the measured variables of the Down syndrome (DS) group.

Variable	*N*	Mean	Median	SD	IQR	Minimum	Maximum
Age	13	15.08	14.00	5.95	7.00	6.00	25.00
Total brain	13	810.94	830.96	223.21	309.71	548.55	1319.84
White matter	13	323.83	298.76	74.73	74.76	239.39	521.85
Grey matter	13	487.11	532.20	158.09	249.31	305.46	797.99
Cerebrum	13	724.95	743.89	201.23	275.65	485.23	1183.56
Cerebellum	13	79.32	78.00	21.45	31.48	55.34	127.30
Brainstem	13	12.25	12.36	2.38	2.12	9.19	18.35
Hippocampus	13	4.48	4.29	.91	1.14	3.34	6.71
Dentate gyrus	13	.19	.15	.09	.08	.07	.36
Ammon's horn	13	2.90	2.77	.64	.69	2.10	4.37
Subiculum	13	1.39	1.30	.32	.37	1.04	2.12
Frontal lobe	13	126.15	130.52	53.91	79.25	65.27	240.27
Parietal lobe	13	71.57	82.64	27.52	47.64	38.15	107.16
Angular gyrus	13	14.72	13.04	4.27	4.26	9.52	24.75
Supramarg. gyrus	13	9.30	8.54	1.99	2.30	6.23	13.48
Temporal lobe	13	64.40	56.95	18.04	17.55	40.55	100.18
Fusiform gyrus	13	13.70	15.55	6.75	10.76	5.97	24.67
*S*. temp. Gyrus	13	6.61	5.75	2.33	3.10	3.52	10.97
Occipital lobe	13	54.59	49.06	16.00	20.16	33.94	91.73
Parahippoca. gyrus	13	4.70	4.20	2.25	3.51	2.12	7.96
Total IQ	13	65.08	65.00	7.52	13.00	53.00	78.00
Nonverbal IQ	13	68.00	65.00	10.82	16.00	54.00	89.00
Verbal IQ	13	63.08	64.50	6.35	7.75	52.00	72.00
Working memory	13	60.38	58.00	7.15	13.00	53.00	74.00
Total language	13	58.92	61.70	17.41	23.40	31.60	87.80
Expressive language	13	66.36	56.30	30.63	45.30	20.80	120.60
Receptive language	13	73.64	71.30	33.93	50.90	28.20	132.10
Fluid reasoning	13	76.15	79.00	13.77	22.00	54.00	97.00
Knowledge	13	74.23	76.00	14.27	16.00	52.00	103.00
*Q*. reasoning	13	62.92	61.00	8.27	8.00	53.00	79.00
*V*. *S*. processing	13	61.08	58.00	7.59	10.00	53.00	74.00

Abbreviations: IQ, intelligence quotient; IQR, interquartile range; *N*, the number of studied samples; parahippoca. gyrus, parahippocampal gyrus; *Q*. reasoning, quantitative reasoning; *S*. temp. gyrus, superior temporal gyrus; SD, standard deviation; supramarg. gyrus, supramarginal gyrus; *V*. *S*. processing, visuospatial reasoning.

### 2.8 Luttas language development test scale

The test aims to assess the child's language development level and extract the child's expressive linguistic and receptive linguistic age. It can define the child's language weaknesses and strengths and develop an appropriate rehabilitation program for language development for each child separately. This test is available for use individually by assessing the child's capacity to recognize the internal language, name and identify implicit groups, understand, and express object functions, understand and communicate linguistic context, and identify expression only for the melodic and pragmatic structure. The test is available in Arabic and developed in Egypt but is suitable for use in other countries, such as Sudan. The test measures expressive, receptive, and overall language scores (see Tables 1 and 2 ) (http://luttas.net/Elct.HTML).

### 2.9 Data analyses

We conducted this investigation as a pilot study. Therefore, we conducted a purely exploratory data evaluation, and the *p*‐values obtained are interpreted descriptively and have a confirmatory value. We used *t*‐tests to compare continuous variables between the control and DS groups and report *p*‐values with FDR correction (see Table 3). Normality was assessed neither with statistical tests such as Shapiro–Wilk nor histograms, as the sample sizes per group were small (*N* = 12, *N* = 13 in the control and DS groups, respectively). However, it is possible to assume that the continuous variables measured derive from normally distributed random variables due to their nature.

**TABLE 3:** Univariate comparison of the means of the Down syndrome (DS) and control groups with Welch's *t*‐tests, FDR correction *p*‐values, and corresponding confidence intervals (CI) estimates of the group mean difference reported.

Variable	*p*‐Value	CI min	CI max
Nonverbal IQ	.019	18.659	44.841
Fluid reasoning	.019	13.213	38.479
Total brain volume	.039	38.190	397.412
White matter	.039	16.060	145.151
Cerebellum	.039	8.070	44.818
Brainstem	.039	.619	5.708
Subiculum	.039	.075	.641
Angular gyrus	.039	1.006	8.523
Supramarginal gyrus	.039	.893	7.816
Temporal lobe	.039	7.657	42.501
Total IQ	.039	16.838	56.508
Verbal IQ	.039	15.074	66.259
Knowledge	.039	10.522	69.016
*Q*. reasoning	.039	13.093	57.561
*V*. *S*. processing	.039	14.363	62.983
Hippocampus	.041	.168	1.923
Cerebrum	.043	27.197	352.864
Working memory	.046	7.100	64.131
Superior t. gyrus	.055	.348	8.114
Grey matter	.058	7.216	267.170
Occipital lobe	.058	.720	29.571
Frontal lobe	.060	1.290	86.290
Ammon's horn	.072	−.015	1.257
Expressive language	.142	−22.238	136.015
Total language	.149	−16.351	87.755
Parietal lobe	.175	−7.132	43.189
Dentate gyrus	.199	−0.033	.166
Receptive language	.212	−30.337	106.660
Fusiform gyrus	.443	−3.120	6.895
Parahippocampus *G*.	.443	−1.058	2.341

Abbreviations: CI max, confidence interval maximum; CI min, confidence interval minimum; IQ, intelligence quotient; parahippocampus *G*., parahippocampus gyrus; *Q*. reasoning, quantitative reasoning; superior t. gyrus, superior temporal gyrus; *V*. *S*. processing, visuospatial processing.

To assess the relationship between structural brain region volumes and neuropsychological test scores, we used Spearman's rank correlation and reported a visual representation of the correlation matrix. We used the FDR multiple comparison procedure in the analysis. We defined a significant difference to have a *p*‐value of less than .05 for *t*‐tests. All analyses were performed with R (R Core Team 2021) using the packages dplyr and corrplot (Wei et al., 2017; Wickham et al., 2020).

## 3 RESULTS

We presented the demographic data (age, sex, and weight at birth) and results of cognitive and language tests (total IQ, total language, and working memory) in Tables 4 and 5.

**TABLE 4:** Description of the study cohort of Down syndrome (DS) participants, including non‐PHI‐related characteristics of each participant.

Participants	Age in years	Sex	Weight at birth	Total IQ	Total language	Working memory
**1**	6	Male	Normal	73	41.6	66
**2**	8	Male	Normal	68	44	58
**3**	10	Female	Normal	72	84	69
**4**	11	Male	1.5 kg < normal	57	31.6	53
**5**	12	Female	Normal	78	67.4	74
**6**	14	Female	Normal	53	61.8	53
**7**	14	Male	Normal	64	54.7	58
**8**	15	Female	Normal	67	46.6	53
**9**	17	Male	Normal	62	61.7	61
**10**	18	Male	Normal	56	42.3	55
**11**	22	Male	1.5 kg	72	87.8	66
**12**	24	Female	Less < normal	65	77	66
**13**	25	Male	Normal	59	65.5	53

Abbreviation: IQ, intelligence quotient.

**TABLE 5:** Description of the study cohort of control participants, including non‐PHI‐related characteristics of each participant.

Participants	Age in years	Sex	Weight at birth	Total IQ	Total language	Working memory
**1**	4	Male	1.5 kg < normal	96	52.6	76
**2**	6	Female	Normal	102	65	78
**3**	8	Male	Normal	105	72	81
**4**	9	Male	Normal	96	66	75
**5**	11	Male	1.5 kg < normal	57	31.6	53
**6**	11	Female	Normal	112	90.3	103
**7**	12	Male	Normal	110	88	95
**8**	14	Male	Normal	113	135.5	118
**9**	17	Female	Normal	107	112	110
**10**	18	Male	Normal	115	121	116
**11**	20	Male	Normal	86	99.7	87
**12**	24	Female	Normal	120	140	130

Abbreviation: IQ, intelligence quotient.

### 3.1 Neuroanatomy of Down syndrome

**FIGURE 1:**
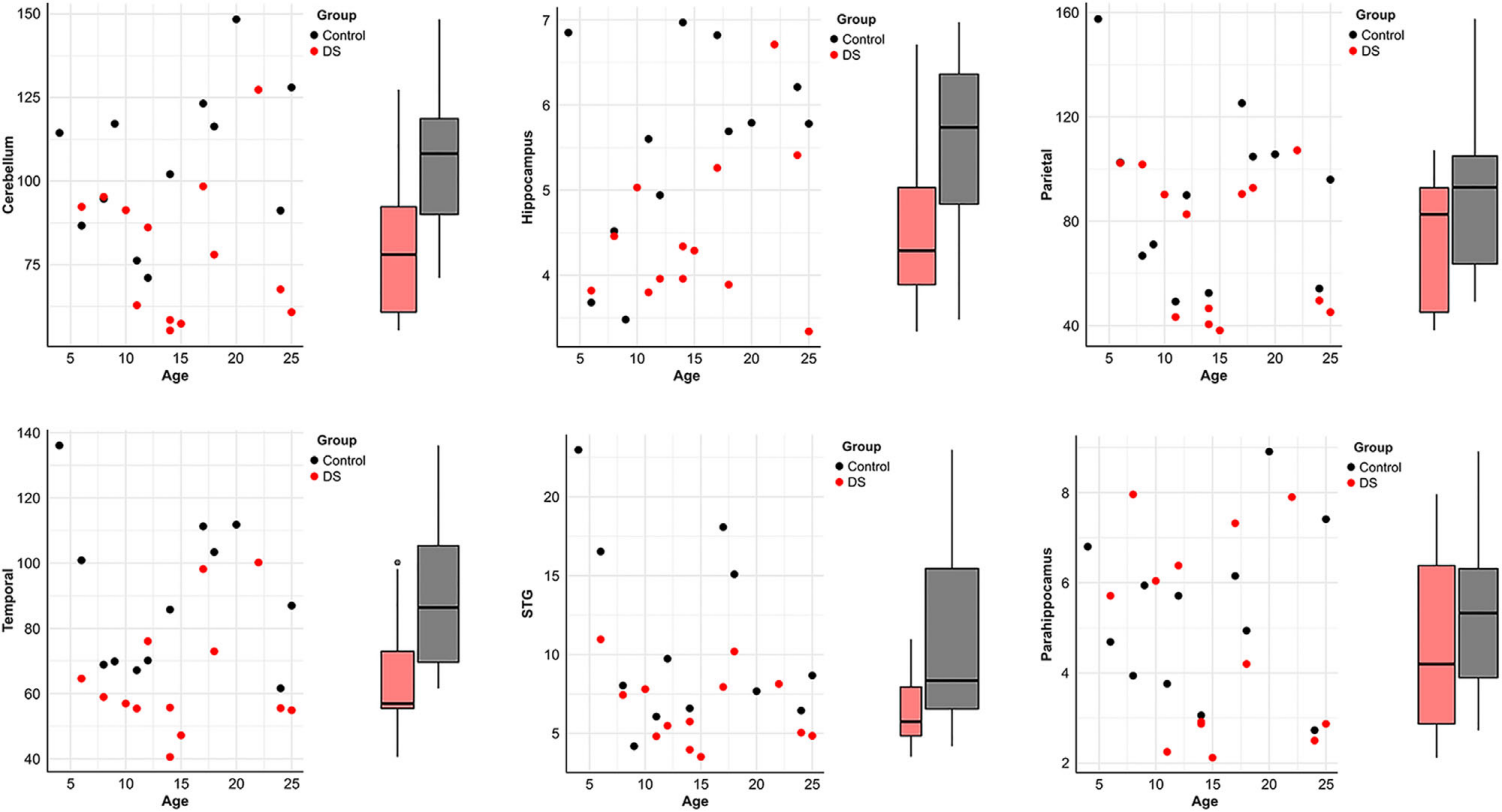
Univariate data visualizations comparing cerebellar, hippocampal, parietal lobe, temporal lobe, superior temporal gyrus (STG), and parahippocampal gyrus volumes in the Down syndrome (DS) and control groups with age control.

Tables 1 and 2 show that the DS participants’ mean total brain volume was 20% smaller than the control group's (*p*‐value = .03). Some regions also showed significant mean group differences, such as the white matter (*p*‐value = .03), cerebellum (*p*‐value = .03), brainstem (*p*‐value = .03), subiculum (*p*‐value = .03), angular gyrus (*p*‐value = .03), supramarginal gyrus (*p*‐value = .03), temporal lobe (*p*‐value = .03), hippocampus (*p*‐value = .04), and cerebrum (*p*‐value = .04). Other regions, such as the STG (*p*‐value = .05), gray matter (*p*‐value = .05), occipital lobe (*p*‐value = .05), frontal lobe (*p*‐value = .06), and Ammon's horn (*p*‐value = .07), approached significance in the difference between the means of the two compared groups. The parietal lobe (*p*‐value = .1), dentate gyrus (*p*‐value = .1), fusiform gyrus (*p*‐value = .4), and parahippocampal gyrus (*p*‐value = .4) showed no significant difference (see table). Significant age‐related changes in different brain regions in the DS or control groups were observable (see Figure 1).

### 3.2 Neuropsychology of Down syndrome

Tables 1 and 2 indicate that the mean total language score in the DS sample was 40% lower than in the control group. Nevertheless, the mean difference between the two groups is insignificant (*p*‐value = .1). The mean expressive language score in the DS group was 50% lower than in the control group, but the mean difference between the two groups was insignificant (*p*‐value = .1). The mean receptive language score in the DS group was 35% lower than that in the control group, and the mean difference in this score between the two groups was insignificant (*p*‐value = .2). Tables 1 and 2 also present the Stanford–Binet intelligence scale (fifth edition) mean scores in the DS and control groups. These scores included total IQ, which was 36% lower in the DS group than in the control group (*p*‐value = .03). The total IQ has two divisions: nonverbal IQ, which is lower in the DS group by 32% than in the control group (*p*‐value = .01), and verbal IQ, which is also lower in the DS group by 40% than in the control group (*p* = .03). Other tests’ mean scores, such as fluid reasoning, are low in DS by 25% than control (*p*‐value = .01), and knowledge is more deficient in DS by 35% than in the control group (*p*‐value = .03). Quantitative reasoning is low in DS by 36% than in the control group (*p*‐value = .01), visuospatial processing is low in DS by 39% than in the control group (*p*‐value = .03), and working memory is also low in DS by 37% than the control group (*p* = .04).

### 3.3 Association between neuroanatomy and neuropsychology of Down syndrome

#### 3.3.1 Correlating total brain, parietal lobe, and temporal lobe volumes with cognitive and language scores

Using the Spearman correlation test, we correlated DS participants’ total brain volume and IQ, working memory, and global language scores. We observed a significant correlation between the whole‐brain volume and working memory (*r* = .68, *p*‐value = .01). The correlation between the total brain volume and global IQ approached significance (*r* = .53, *p*‐value = .06). The correlation between the whole‐brain volume and overall language scores was insignificant.

For DS participants, we also correlated parietal lobe volume with total IQ, working memory, whole language, and visuospatial processing scores. We discovered a significant correlation between the parietal lobe, working memory (*r* = .62, *p*‐value = .02), and visuospatial processing (*r* = .55, *p*‐value = .04). The correlation between the parietal lobe and total IQ approached significance (*r* = .49, *p*‐value = .08). We observed no significant correlation between the parietal lobe and whole language and other language scores.

We correlated temporal lobe volume and total IQ, working memory, whole language, and visuospatial processing scores for DS participants. We found a significant correlation between the temporal lobe and working memory (*r* = .68, *p*‐value = .009). The correlation between the temporal lobe and total IQ approached significance (*r* = .48, *p*‐value = .09), and there was no significant correlation between the temporal lobe and whole language and visuospatial processing.

#### 3.3.2 Correlating superior temporal gyrus, angular gyrus, supramarginal gyrus, and fusiform gyrus volumes with cognitive and language scores

We correlated STG volume, global IQ, working memory, and whole language scores for DS participants. We observed a significant correlation between the STG and working memory (*r* = .57, *p*‐value = .04) and no significant correlation between the STG and total IQ and total language.

For DS participants, we correlated angular gyrus volume with total IQ, working memory, and whole language scores. We observed a significant correlation between the angular gyrus and working memory (*r* = .55, *p*‐value = .04) and no significant correlation between the angular gyrus and total IQ and total language.

We correlated DS participants’ supramarginal gyrus volume with global IQ, working memory, and whole language scores. We observed no significant correlation between the supramarginal gyrus and any of these scores.

We correlated the DS participants’ fusiform gyrus volume and total IQ, working memory, and whole language scores. We observed a significant correlation between the fusiform gyrus and global IQ (*r* = .60, *p*‐value = .02) and working memory (*r* = .73, *p*‐value = .003) and no significant correlation between the fusiform gyrus and total language.

We correlated DS participants’ fusiform gyrus volume, fluid reasoning, knowledge, quantitative reasoning, and visuospatial processing scores. We observed a significant correlation between the fusiform gyrus and visuospatial processing (*r* = .65, *p*‐value = .01). The correlation between the fusiform gyrus and other scores was insignificant.

#### 3.3.3 Correlating hippocampus, parahippocampal gyrus, and hippocampal subregion volumes with cognitive and language scores

We correlated hippocampal volume, whole IQ, working memory, expressive language, and total language scores for DS participants. We discovered a significant correlation between the hippocampus and whole language (*r* = .59, *p*‐value = .03), and the correlation between the hippocampus and working memory (*r* = .51, *p*‐value = .07) and expressive language (*r* = .51, *p*‐value = .07) approached significance. The correlation between the hippocampus and total IQ was insignificant.

We correlated parahippocampal gyrus volume with the DS participants’ whole IQ, working memory, and global language scores.

We observed a significant correlation between the parahippocampal gyrus and working memory (*r* = .57, *p*‐value = .04). There was no significant correlation between the parahippocampal gyrus and total IQ and whole language scores.

We correlated DS participants’ hippocampal subregion (dentate gyrus) volume and total IQ, working memory, and whole language scores. We discovered a significant correlation between the dentate gyrus and whole language (*r* = .64, *p*‐value = .01) and no significant correlation between the dentate gyrus and working memory and total IQ.

We correlated dentate gyrus volume with DS participants’ fluid reasoning, knowledge, quantitative reasoning, and visuospatial processing scores. We found no significant correlations between the dentate gyrus and these scores.

We correlated DS participants’ hippocampal subregion (Ammon's horn) volume and total IQ, working memory, and whole language scores. We observed that the correlation between Ammon's horn and global language approached significance (*r* = .50, *p*‐value = .07) and that there was no significant correlation between Ammon's horn and other scores.

We correlated Ammon's horn volume, fluid reasoning, knowledge, quantitative reasoning, and visuospatial processing scores for DS participants. We found no significant correlation between Ammon's horn and these scores.

We correlated hippocampal subregion (subiculum) volume, total IQ, working memory, and whole language. We found a significant correlation among the subiculum, global IQ (*r* = .59, *p*‐value = .03), and working memory (*r* = .61, *p*‐value = .02). There was no significant correlation between the subiculum and total language.

We correlated subiculum, fluid reasoning, knowledge, quantitative reasoning, and visuospatial processing. We found a significant correlation between the subiculum and visuospatial processing (*r* = .62, *p*‐value = .02). We observed that the correlation between the subiculum and quantitative reasoning approached significance (*r* = .49, *p*‐value = .08). There was no correlation between the subiculum and other scores.

## 4 DISCUSSION

### 4.1 Significant neuroanatomical and neuropsychological findings in Down syndrome

**FIGURE 2:**
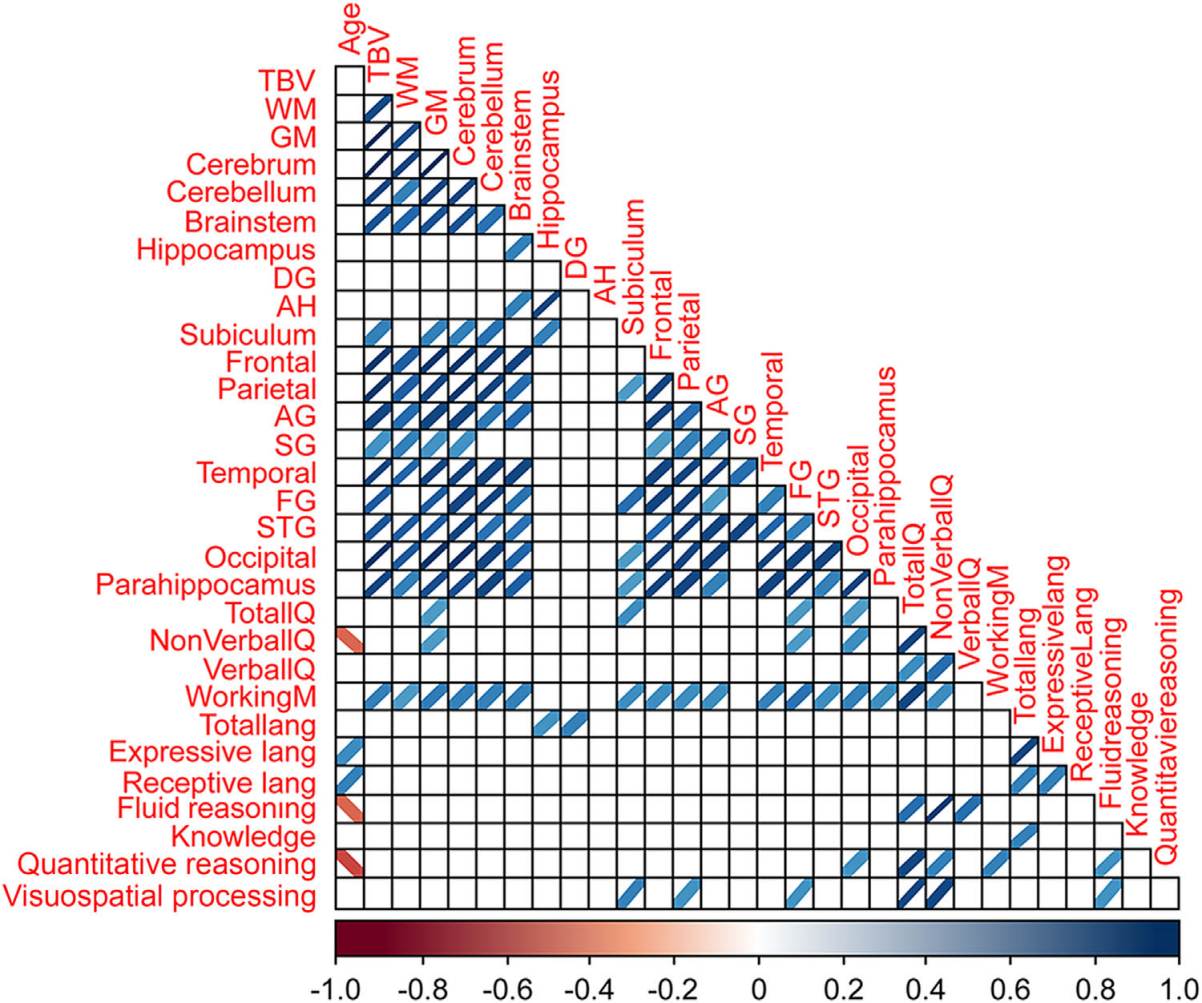
Correlation matrix visualization within the Down syndrome (DS) group (Spearman's rank correlation matrix visualization). Note that this figure depicts only the significant correlations. The color of the ellipse and the direction it leans correspond to the magnitude and direction of the correlation coefficient. TBV, total brain volume, WM, white matter, GM, gray matter, DG, dentate gyrus, AH, Ammon's horn, AG, angular gyrus, SG, supramarginal gyrus, FG, fusiform gyrus, STG, superior temporal gyrus, IQ, intelligence quotient.

**FIGURE 3:**
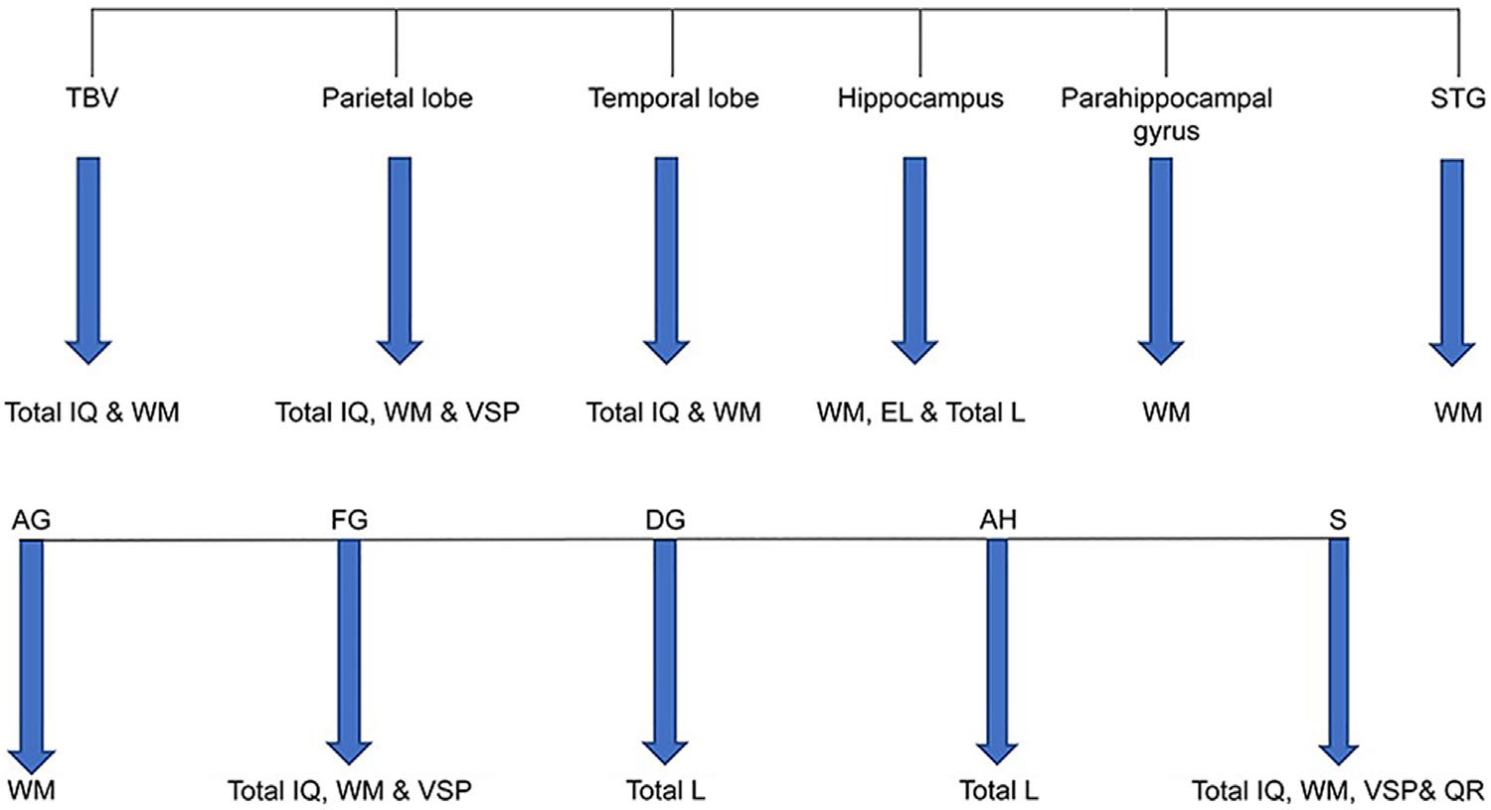
Schematic illustration of correlations between brain regions and subregion volumes and cognitive and language scores. TBV, total brain volume, STG, superior temporal gyrus, IQ, intelligence quotient, WM, working memory, VSP, visuospatial processing, E L, expressive language AG, angular gyrus, FG, fusiform gyrus, DG, dentate gyrus, AH, Ammon's horn, S, subiculum, E L, expressive language, IQ, intelligence quotient, WM, working memory, VSP, visuospatial processing, QR, quantitative reasoning.

**FIGURE 4:**
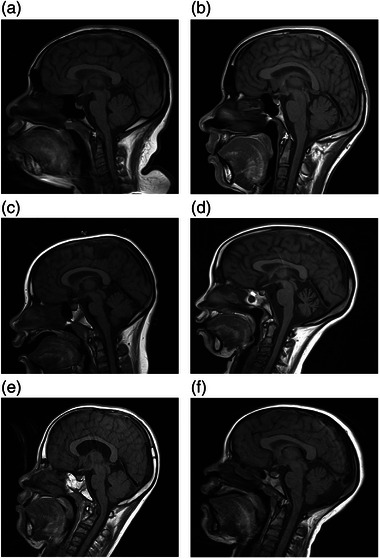
Sagittal T1/SE magnetic resonance imaging (MRI) view of (a) a 25‐year‐old adult male with Down syndrome (DS), (b) a 25‐year‐old male control participant, (c) a 12‐year‐old female with DS, (d) a 12‐year‐old female control participant, (e) a 14‐year‐old male with DS, and (f) a 14‐year‐old female with DS for comparison between DS and control participants as well as between DS males and females.

**FIGURE 5:**
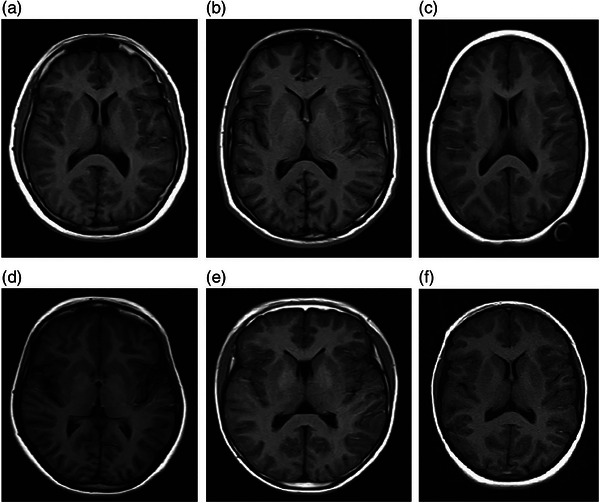
Axial T1/SE magnetic resonance imaging (MRI) view of (a) a 25‐year‐old adult male with Down syndrome (DS), (b) a 25‐year‐old male control participant, (c) a 12‐year‐old female with DS, (d) a 12‐year‐old female control participant, (e) a 14‐year‐old male with DS, and (f) a 14‐year‐old female with DS for comparison between DS and control participants as well as between DS males and females.

We investigated regional brain volumes in children and adults with DS to identify the structural neuroanatomical abnormalities related to their cognitive features. We collected imaging data from the participants with DS and the controls and correlated the MRI data of the DS group with cognitive functions evaluated using neuropsychological battery assessments. We applied different neuropsychological tasks to investigate cognitive domains, such as global cognition, memory, and language, and we correlated the results to various regional brain volumes (see Figures 2 and 3). Our results confirmed that persons with DS had reduced total brain, cerebrum, cerebellum, brainstem, hippocampus, frontal lobe, parietal lobe, temporal lobe, and occipital lobe volumes and greater parahippocampal gyrus volume than controls (see Figures 1, 4, and 5). These findings are consistent with previous neuropathological and neuroimaging studies (Aylward, Habbak, et al., 1997; Hamner et al., 2018; Jernigan et al., 1993; Kesslak et al., 1994; Menghini et al., 2011; Mullins et al., 2013; Pinter, Eliez, et al., 2001; White et al., 2003).

Consistent with the findings by Kesslak et al. (1994), Raz et al. (1995), and White et al. (2003) of larger parahippocampal gyrus volume in people with DS compared to controls, our study also showed greater parahippocampal gyrus volume in DS than in controls (see Figure 1). Furthermore, our results indicated reduced white matter and gray matter volumes in the total brain, hippocampal subregions (dentate gyrus, Ammon's horn, and subiculum), angular gyrus, supramarginal gyrus, fusiform gyrus, and STG in DS participants compared to controls. Our study correlational analysis did not indicate any age‐related changes in brain areas in the DS or control groups. The neuropsychological profile of persons with DS showed deficits in various cognition and language domains in our study group. Our study results confirmed the findings of previous studies that impairments in expressive language are more remarkable than deficits in receptive language (Abbeduto et al., 2001; Pulina et al., 2019). Our study group showed a mean IQ of 65, consistent with previous studies that confirmed that most people with DS have an IQ between 30 and 70 (Abbeduto et al., 2007). Our DS group showed a higher nonverbal than verbal IQ, contrasting with the results of a report by Evans and Uljarević (2018). This study described that children and adolescents with DS have a higher verbal IQ than nonverbal (assessed with the Stanford–Binet intelligence scale fourth edition) (Evans & Uljarević, 2018). We also confirmed deficits in working memory, consistent with Couzens and Haynes’ findings (Couzens et al., 2012). Although the cognitive profile of DS shows relative strength in visuospatial processing skills (Pinter, Eliez, et al., 2001), our study group showed impairment in visuospatial processing compared to controls (see Tables 1 and 2).

Additionally, this deficit in visuospatial processing skills is remarkable compared to other verbal and nonverbal abilities. A review by Yang et al. (2014) also described visuospatial working memory as a weak area in DS. Other domains, such as fluid reasoning, knowledge, and quantitative reasoning, showed impairment in our study group (see Table 2). Previous research studies also reported similar findings (Barisnikov & Lejeune, 2018). Some study findings revealed divergent development patterns of these domains, with crystallized abilities as verbal tasks, which refer to accumulating knowledge, facts, and skills acquired throughout life, increasing until approximately 20 years of age and then gradually declining. Fluid skills as visuospatial skills, which refer to the ability to reason and think flexibly, followed a distinct trajectory. They show significant development in the early years, followed by continual growth and no decline (as demonstrated for the different components) until at least 30‐year old (Couzens et al., 2011; Grieco et al., 2015; Pulina et al., 2019).

### 4.2 Correlations between different brain regions and language and memory functions in Down syndrome

Raz et al. (1995) found no relationship between total brain volume and cognitive variables in DS. Nevertheless, our results showed an association between total brain volume reduction and deficits in full‐scale IQ and working memory (see Figure 3). This finding confirms previous reports that found a positive association between brain volume and intelligence in the general population (McDaniel, 2005; Ritchie et al., 2015). Evidence exists for the association between the parietal lobe and visuospatial processing skills in DS (Pinter, Eliez, et al., 2001). This relationship depends on the preserved parietal lobe volume finding associated with previous reports’ relative strength in visuospatial processing (Pinter, Eliez, et al., 2001). However, our results of reduced parietal lobe volume and impaired visuospatial processing with a positive correlation contrast these reports (see Figure 3).

Additionally, we observed a correlation between the reduction in parietal lobe volume and deficits in working memory in DS, which Menghini et al. (2011) confirmed (see Figure 3). Our study could not confirm the association between reduced parietal lobe volume and deficits in linguistic abilities in DS. No similar research reported the relationship between reduced parietal lobe volume and deficits in language skills in DS. The relationship between decreased parietal lobe volume and low total IQ approached significance. Nevertheless, there is evidence of a correlation between the parietal lobe and intelligence in the general population (Yoon et al., 2017). Our study results confirmed the association between temporal lobe volume reduction and deficits in working memory in DS (see Figure 3). This finding ensures the temporal lobe's role in memory function in DS (Galaburda & Schmitt, 2003; Hamadelseed et al., 2022; Menghini et al., 2011; Mullins et al., 2013; Pennington et al., 2003; Vicari, 2006). Our results could not confirm the involvement of the temporal lobe in language deficits in DS.

Nevertheless, in contrast to a study by Pinter, Eliez et al. (2001), which reported larger corrected volumes of temporal lobe volume, our results showed a reduced temporal lobe volume in DS participants (see Figure 3). This result provides neuroimaging evidence for the hypothesis of disproportionately smaller temporal lobe volumes associated with language deficits in DS (Pinter, Eliez, et al., 2001). The relationship between the reduced temporal lobe volume and low total IQ approached significance.

Nevertheless, there is evidence of a correlation between the temporal lobe and intelligence in the general population (Yoon et al., 2017). Our study confirmed the link between hippocampal volume reduction and deficits in language functions and working memory in DS (see Figure 3), which has been reported widely by previous studies (Krasuski et al., 2002; Pennington et al., 2003; Raz et al., 1995). These findings reflect the role of the hippocampus as an essential biomarker for AD and one of the regions severely affected by the neuropathological changes of AD (Aylward et al., 1999; Hamadelseed et al., 2022). We found no significant correlation between age and hippocampal volume. This finding is consistent with previous studies by Raz et al. (1995) and Aylward et al. (1999), who failed to find a correlation between age and hippocampal volume but contrasts with the study by Kesslak et al. (1994), who found a significant correlation between age and hippocampal volume. The age range of these reported studies was between 22 and 50 years, and our study group's age range was between 6 and 25 years. This comparison suggests that the significant decrease in hippocampal volume before age 30 remains stable and decreases later when dementia occurs in people with DS (Aylward et al., 1999). This decrease in hippocampal volume with increased age is related to changes in the neural pathway associated with memory and learning problems that start in infancy and continue throughout childhood (Kates et al., 1997).

The most exciting finding in our study is the greater volume of the parahippocampal gyrus, which agrees with the results of Kesslak et al. (1994), Raz et al. (1995), and White et al. (2003), who reported enlargement of this structure that is severely affected by AD.

The parahippocampal gyrus volume does not follow DS's known neuroanatomical, neurodevelopmental, and pathological pathways. Another interesting finding related to parahippocampal gyrus volume is the association between this structural volume reduction and deficits in working memory (see Figure 3). There was no association between parahippocampal gyrus volume reduction and total IQ and language deficits. This result contrasts with Raz et al. (1995), who found a negative correlation between parahippocampal gyrus volume and IQ in people with DS. Our imaging analysis results significantly support the suggestion of narrowness of the STG (see Figure 1) (Nadel, 1999; Pennington et al., 2003). However, some related studies could not confirm this (Kesslak et al., 1994; Pinter, Eliez, et al., 2001). The STG is part of the language network (Friederici & Gierhan, 2013). It contributes to the perceptual analysis of the speech signal during auditory word processing and the production and comprehension of spoken words (Zevin, 2009). Postmortem investigations of DS revealed aberrant patterns of cortical lamination in the superior temporal neocortex, making the study of the STG abnormalities in DS significant (Golden & Hyman, 1994; Ross et al., 1984). Furthermore, compared to individuals with a developmental delay where linguistic abilities are preserved, people with DS have reduced volumes of temporolimbic structures, indicating that these anomalies may cause the language impairment reported in this syndrome.

Our study indicated a correlation between a reduced STG volume and deficits in working memory (see Figure 3). In contrast with previous reports, we did not find an association between a reduction in STG volume and scores of total IQ and total language and other language scores.

We tried to include other specific brain regions related to deficits in language and memory in DS. These included the temporoparietal junction (e.g., angular and supramarginal gyri) and occipitotemporal structures (e.g., fusiform gyrus), which are parts of the language network (Friederici & Gierhan, 2013; Hamner et al., 2018; Hickok & Poeppel, 2004).

We could not confirm the relationship between angular gyrus volume and language deficits in DS. We found a correlation that approached significance between angular gyrus volume and deficits in working memory in DS (see Figure 3). This result is consistent with reports about the role of the angular gyrus in verbal working memory and other complex cognitive functions in the general population (Seghier, 2013).

The reduced fusiform gyrus volume, also known as the occipitotemporal gyrus, correlates with deficits in total IQ and working memory in DS (see Figure 3).

An interesting finding is an association between reduced fusiform gyrus volume, a parietal lobe subregion, and impaired visuospatial processing skills in DS (Figure 3). This finding supports our link between reduced parietal lobe volume and deficits in visuospatial processing skills in DS. As part of our study of the hippocampal formation, we studied three hippocampal subregions (dentate gyrus, Ammon's horn, and subiculum) to understand their role in the cognitive and language skills of DS. We observed an association between reduced dentate gyrus volume and deficits in total language skills (see Figure 3). Dentate gyrus function in the production of long‐term memory is evident by studying impaired neurogenesis in DS fetuses and Ts65Dn DS mouse models (Contestabile et al., 2007). We found an association between reduced Ammon's horn volume and deficits in the total language in DS (see Figure 3).

Interestingly, reduced subiculum volume is associated with deficits in total IQ, working memory, visuospatial processing, and quantitative reasoning skills in DS (see Figure 3). We suppose that the subiculum plays a significant role in cognition and memory processing in DS compared to other hippocampal subregions. The subiculum plays an essential role in the hippocampal circuit. Nevertheless, little is known about its function, although some reports indicate a critical but ill‐defined role in spatial navigation and mnemonic processing (O'Mara et al., 2001).

### 4.3 Working memory and its relationship with brain areas in Down syndrome

Previous research showed that people with DS have impaired working memory, as evaluated by a digit or word span, compared to groups of mentally age‐matched controls (Vicari, 2006). Working memory is a limited‐capacity mechanism for storing information for later manipulation. Working memory stimulates a sizeable neuronal network, including multiple frontal and parietal areas. The nature of the tasks appears to differentiate these areas. For example, the dorsolateral prefrontal cortex (PFC) and the superior frontal sulcus are engaged in activities requiring information updating and sequencing.

In contrast, the ventral and anterior PFCs are involved in manipulation tasks (Godfrey & Lee, 2018). Vicari (2006) indicated that frontocerebellar and temporal areas are engaged in processing working memory. Menghini et al. (2011) demonstrated the role of the cerebellum and right inferior parietal lobule in working memory. The involvement of the right inferior parietal lobule in working memory has been shown in healthy controls (Colom et al., 2007; Gerton et al., 2004). Müller and Knight (2006) indicated the role of the PFC in working memory. Our study showed the involvement of several brain areas in working memory impairment in DS, such as the temporal lobe (mainly the STG and fusiform gyrus), parietal lobe (particularly the angular gyrus), hippocampus (mainly the subiculum), and parahippocampal gyrus (see Figure 3). There is very little research on working memory skills in DS over the lifetime. Therefore, our study findings support the reported literature about the neuroanatomical areas involved in working memory, adding more details that can predict the onset of dementia in people with DS and the general population.

Executive function comprises working memory, cognitive flexibility, and inhibition and depends on top–down (i.e., goal‐driven) control of distributed processes occurring throughout the brain. The exact behavioral output (i.e., function) depends on the content of the processes being controlled. The PFC is a brain region critical for learning and memory (Alemany‐González et al., 2020; Friedman & Robbins, 2022). A study by Jojoa‐Acosta et al. (2021) found that adults with DS have impaired inhibitory capacity. Another study found that impaired executive function in DS is due to abnormal PFC development (Rowe et al., 2006). Our study results confirmed these conclusions and showed reduced volumes of the frontal lobe regions, including the PFC, and impaired associated cognitive functions such as working memory.

### 4.4 The potential of biomarker analysis in Down syndrome

Biomarker analysis has the potential to help us understand DS better. According to a study published in The Lancet, biomarker criteria can be used to streamline the recruitment of people with DS for clinical trials. Additionally, biomarker characterization will be helpful for future precision medicine approaches and essential for developing effective interventions within this high‐risk population (Head & Ances, 2020).

Another study published in Nature found that phosphorylated tau181 in plasma could be a potential biomarker for AD in individuals with DS (Lleó et al., 2021). This suggestion is important because people with DS are at high risk of developing AD with aging. The diagnosis and treatment trials are hampered by a lack of reliable blood biomarkers (Shinomoto et al., 2019).

### 4.5 Limitations of the current study

Despite the valuable insights gained from this neuroradiological study on individuals with DS, it is important to acknowledge several pitfalls and limitations that may have impacted the interpretation of the findings. These limitations should be considered when interpreting the results and future research directions.

First, the limited study power poses a significant challenge in detecting subtle or minor differences in neuroimaging measures between individuals with DS and controls.

The relatively small population size and inherent difficulties in recruiting an adequate sample of participants with DS restricted our statistical power. Consequently, caution should be exercised when drawing definitive conclusions or generalizing the findings to the broader population of individuals with DS.

A notable limitation arises from the inherent heterogeneity within the DS population. This heterogeneity encompasses variations in cognitive abilities, comorbidities, and developmental trajectories. The diverse nature of this population introduces confounding factors that may have influenced the observed neuroimaging measures, potentially leading to a lack of consistency across the study sample. Consequently, the generalizability of our findings may be limited to specific subgroups within the DS population.

Another significant limitation pertains to the challenges of analyzing MRI images in individuals with DS. The unique anatomical characteristics, potential motion artifacts, or co‐occurring conditions may have introduced variability or inaccuracies in the image analysis. It is crucial to consider these factors when interpreting the obtained neuroimaging results.

Additionally, assessing cognitive functioning in individuals with DS poses challenges due to the limitations of traditional cognitive tests. These tests may not fully capture the diverse cognitive abilities within this population and could be affected by floor or ceiling effects. Consequently, establishing robust correlations between neuroimaging findings and cognitive performance, particularly in individuals without intellectual disabilities, may be challenging.

The age range and developmental factors present another limitation in this study. DS is associated with specific developmental trajectories that differ from typical development. Including participants across a wide age range introduces confounding effects related to age‐dependent brain changes, developmental stages, and the potential influence of interventions or therapies received throughout the life span. Longitudinal studies encompassing a larger sample size and multiple time points would be valuable for capturing developmental trajectories and distinguishing age‐related changes from syndrome‐specific effects.

Furthermore, the lack of longitudinal data is a significant limitation in our study design. Cross‐sectional data provide a snapshot of brain characteristics at a specific time. Still, it limits our ability to determine whether the observed differences are inherent to DS or result from developmental or aging processes. Future studies incorporating longitudinal designs would provide a more comprehensive understanding of the neurodevelopmental trajectory in DS. Lastly, it is crucial to recognize the limited generalizability of our findings. The specific characteristics of our sample, including the age range, cognitive abilities, and recruitment methods, may have introduced selection biases. Consequently, caution should be exercised when extrapolating the results to the broader DS population.

In conclusion, although this neuroradiological study has shed light on structural brain differences in individuals with DS, the pitfalls and limitations discussed above should be considered. Future research endeavors should address these limitations through larger sample sizes, longitudinal designs, and improved methodologies. Such efforts will contribute to a more comprehensive understanding of the neurobiology of DS and its implications for cognitive functioning and development.

## 5 CONCLUSIONS

This study is the first to study and assess the neuroanatomy and neuropsychology of DS in detail using high‐resolution neuroimaging techniques, considering the limitations of previous related studies. Diversity and inclusion of individuals with DS and matched control persons without intellectual disabilities from various socioeconomic backgrounds, races, and ethnicities are now the principal focus of clinical and translational research (Fidler et al., 2022). Our study represents a largely age‐matched, largely sex‐matched, comprehensive MRI, and behavioral study of children with DS from an African nation with Arabic as the primary language. Our results confirm earlier reports regarding overall patterns of brain volumes in individuals with DS and provide new evidence for abnormal volumes of specific regional and subregional brain volumes associated with language and memory domains. Despite the small number of our study participants, we found different patterns of neuroanatomic abnormalities in DS. The difficulty in recruiting children and adults with DS and convincing their families to participate in the study, the cost, and the time‐consuming nature of radiological and psychological examinations limited the number of participants. Additionally, we could not have children under 5 years of age, not due to the rarity of the proposed participants but because the skills of the children are not enough to perform the neuropsychological assessment and respond to its content. Although understanding the neuropathological nature of DS deserves to be pursued, studying the relationship between abnormal neuroanatomy and deficits in memory and language is of greater scientific and practical importance. The findings of this study indicate that the brains of individuals with DS show a well‐defined pattern of abnormalities. The correlational analysis presented in this study's results section provides excellent evidence representing firm conclusions. Within the studied group of developmentally delayed individuals, the degree of the global reduction in brain volume predicts the general level of intellectual performance and memory function.

Additionally, decreased parietal lobe volume may significantly predict cognitive disabilities in DS, especially those associated with visuospatial processing skills. Similarly, reduced volumes of the temporal lobe and hippocampus may significantly predict cognitive functions in DS, especially those associated with memory and language skills. The parahippocampal gyrus volume was more significant in persons with DS than in normal controls and was related to deficits in working memory function. Therefore, parahippocampal gyrus enlargement, also indicated by independent researchers (Kesslak et al., 1994; Raz et al., 1995; White et al., 2003), and its specificity to DS, when compared with normal aging and AD, was also reported by our study. Other neuroanatomic abnormalities could also be important markers because of their association with cognitive deficits. These markers include the STG and its association with working memory.

Additionally, regions, such as the angular gyrus, supramarginal gyrus, and fusiform gyrus, are essential in understanding the language network and its association with memory functions. Hippocampal subregions (dentate gyrus, Ammon's horn, and subiculum) are crucial to understanding the role of hippocampal formation and its association with the memory domain. A study by Kłosowska et al. (2022) suggested that the height percentile of children with DS correlates with their IQ. These observations may be valuable in expanding our understanding of the cognitive function of people with DS when correlating growth parameters, such as height, to the imaging, and IQ test results in our analysis. However, problems with data availability make this impossible in the current study and can be considered in future studies. A more extensive and longitudinal study is needed to study neuroanatomical and behavioral changes with increasing age. This recommended study should include interventional rehabilitation programs to observe the effects of these methods to improve cognitive skills or prevent a more significant decline with time. From a practical standpoint, these data can provide educational psychologists and teachers invaluable information for developing rationally grounded interventions to understand and alleviate these individuals’ learning difficulties and social problems.

## CONFLICT OF INTEREST STATEMENT

The authors declare that they have no conflicts of interest.

### PEER REVIEW

The peer review history for this article is available at https://publons.com/publon/10.1002/brb3.3186

## Data Availability

The data supporting this study's findings are available from the corresponding author upon reasonable request.
